# Optimising the scan delay for arterial phase imaging of the liver using the bolus tracking technique

**DOI:** 10.2349/biij.7.2.e12

**Published:** 2011-04-01

**Authors:** RS Chan, G Kumar, BJJ Abdullah, KH Ng, A Vijayananthan, H Mohd. Nor, YW Liew

**Affiliations:** Department of Biomedical Imaging, Faculty of Medicine, University of Malaya, Kuala Lumpur, Malaysia

**Keywords:** Arterial phase imaging of the liver, bolus tracking technique

## Abstract

**Objective:**

To optimize the delay time before the initiation of arterial phase scan in the detection of focal liver lesions in contrast enhanced 5 phase liver CT using the bolus tracking technique.

**Patients and Methods:**

Delay - the interval between threshold enhancement of 100 hounsfield unit (HU) in the abdominal aorta and commencement of the first arterial phase scan. Using a 16 slice CT scanner, a plain CT of the liver was done followed by an intravenous bolus of 120 ml nonionic iodinated contrast media (370 mg I/ml) at the rate of 4 mL/s. The second phase scan started immediately after the first phase scan. The portal venous and delay phases were obtained at a fixed delay of 60 s and 90 s from the beginning of contrast injection. Contrast enhancement index (CEI) and subjective visual conspicuity scores for each lesion were compared among the three groups.

**Results:**

84 lesions (11 hepatocellular carcinomas, 17 hemangiomas, 39 other hypervascular lesions and 45 cysts) were evaluated. CEI for hepatocellular carcinomas appears to be higher during the first arterial phase in the 6 seconds delay group. No significant difference in CEI and mean conspicuity scores among the three groups for hemangioma, other hypervascular lesions and cysts.

**Conclusion:**

The conspicuity of hepatocellular carcinomas appeared better during the early arterial phase using a bolus tracking technique with a scan delay of 6 seconds from the 100 HU threshold in the abdominal aorta.

## INTRODUCTION

Multiphase CT improves the sensitivity of liver lesions detection. MRI, despite being recognised to be superior in characterising focal liver lesions, remains a secondary tool due to limited resources in most centres in Malaysia.

Approximately 30% of focal liver lesions are hypervascular and detectable exclusively on the arterial phases [1,2]. The early arterial phase of liver CT provides the best enhancement of hepatic arterial vessels and arterial-portal venous shunts [3]. Hypovascular liver metastases are most conspicuous on the portal venous phase [4,5]. Optimising the scan delay for the arterial phases is, therefore, important to improve the detection of hypervascular liver lesions.

Some researchers found triple-phase CT superior to dual-phase imaging in the detection and characterisation of hepatocellular carcinoma (HCC) [1,6]. Foley et al. and Francis et al. found that hypervascular hepatic tumours are best enhanced on the late arterial phase with 24.5 to 35 seconds delay from the initiation of contrast injection [7,8]. Murakami et al., however, recommended double arterial phase scan to improve the detection of hypervascular HCCs [9].

Other than the scan delay, the conspicuity of liver lesions are also affected by the lesion’s size, the patient’s body weight, cardiac output, blood pressure and pulse rate, the contrast volume, concentration and injection rate, and the scan parameters. This study aims to optimise the scan delay of contrast-enhanced 5-phase CT of the liver using a 16-slice MDCT and a bolus tracking technique to improve the detection of hypervascular liver lesions.

## PATIENTS AND METHODS

This is a prospective study with approval from the ethical review committee, evaluating all patients who underwent 5-phase CT of the liver from February to October 2008 for suspected space occupying liver lesion based on clinical, laboratory or ultrasound findings.

The inclusion criteria were: age above 15 years, serum creatinine of less than 1.5 mg/dL and no contraindications to iodinated contrast material. Patients were excluded based on several criteria which may affect liver vascularity, i.e. patients with diffuse liver lesions or lesion larger than 15 cm in diameter; previous radiofrequency ablation (RFA), transarterial chemoembolisation (TACE), percutaneous transhepatic cholangiography (PTC) or transjugular intrahepatic portosystemic shunt (TIPS); portal venous thrombosis, portal hypertension, hepatic venous thrombosis, inferior vena caval thrombosis or hepatic arterial portal shunt; technical failure related to contrast medium injection, breath-holding or machine problem during CT examination.

All patients fasted for at least 6 hours prior to the CT examination. Contraindications to contrast injection were ruled out. Written informed consent was obtained. Patients were given 500 ml of plain water as oral contrast. An 18G branula was placed at the patients' antecubital vein.

The study was performed using a GE Lightspeed 16­MDCT scanner (Milwaukee, Wisconsin, USA) at the following settings: rotation time, 0.5 second; beam collimation, 16 × 0.625 mm; section thickness and intervals, 5.0 mm; pitch ratio, 1.375:1; table speed 13.75 mm/s; scan time, 0.5 s, gantry tilt, 0.0; voltage, 120 kVp; and tube current, 300 mAs. A bolus-tracking technique with automated scan-triggering software (SmartPrep, GE Healthcare) was used. A circular region of interest (ROI) was placed within the abdominal aorta just above the celiac axis level. The threshold CT value was preset at 100 HU. One of the three protocols with a scan delay of 3 seconds, 6 seconds or 9 seconds (from the time when threshold enhancement was detected to the beginning of the early arterial phase scan) was pre-selected for each patient randomly prior to the examination.

All patients underwent a non-contrast enhanced liver CT (Phase 1). A dual-syringe injector system (Stellant Medrad) was used for intravenous administration of 120 ml non-ionic contrast media (Ultravist 370 mg I/mL) at 4 mL/s followed by 30–40 ml of saline chaser bolus. Real-time low-dose serial monitoring studies began 13 seconds from the beginning of contrast injection. The early arterial phase scan (Phase 2) was triggered using one of the three scan protocols with a different time delay when the threshold enhancement was detected. Helical scan for the early arterial phase of the liver was performed in a cephalocaudal direction. The late arterial phase scan (Phase 3) started immediately following the early arterial phase scan, in a caudocephalic direction. The portal venous (Phase 4) and delayed scans (Phase 5) started at a fixed delay of 60 seconds and 90 seconds respectively, from the beginning of contrast injection. Patients were instructed to hold their breath in full inspiration during each scan phase (Figure 2).

**Figure 1:**
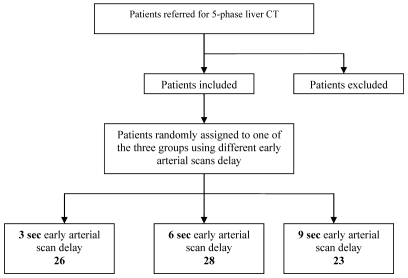
Study design.

**Figure 2:**
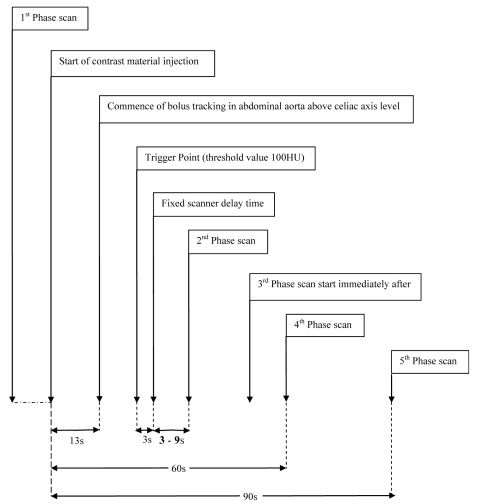
The CT protocols for the 3 patient groups using the bolus tracking method with the scan delays of 3s, 6s and 9s.

Post-processing images were reconstructed into a 34–50 cm display FOV depending on the patients’ physique, at 1.250 cm sections and a window of 400:40 HU. Image analysis was performed at a single workstation (Advantage Window 4.2, GE Healthcare) on an LCD monitor with a spatial resolution of 1280 × 1024 pixels (Radiforce R22, Eizo).

## IMAGE ANALYSIS

The conspicuity of each liver lesion was evaluated quantitatively and qualitatively on each scan phase. In patients with more than three lesions, the largest three lesions were assessed.

### Quantitative image analysis

The attenuation of lesion was measured with a circular region of interest (ROI) by a radiologist who was blinded to the scan protocols, without changing the preset window (400:40 HU), with an attempt to maintain an ROI area of 50 mm^2^ at the centre or the most homogenously enhancing part of the lesion and with care to avoid regions of rim enhancement, tumour capsule, necrosis, calcifications or vessels. The attenuation of the background liver parenchyma was measured in two areas adjacent to the lesion and averaged, using a constant ROI area of 200 mm^2^, at least 1 cm away from the edge of the lesion to nullify the risk of encountering fibrosis [19], excluding visible vessels, bile ducts and artefacts. The conspicuity of a lesion was expressed using the Contrast Enhancement Index (CI):

$$CI=\frac{\begin{array}{l} Lesion\;attenuation-Average\;background\;liver\\ \;\;\;\;\;\;\;\;\;\;\;\;\;\;\;\;\;\;\;\;\;\;\;\;\;\;\;\;\;\;\;parenchymal\;attenuation \end{array}}{Average\;background\;liver\;parenchymal\;attenuation}$$

### Qualitative image analysis

**Figure 3:**
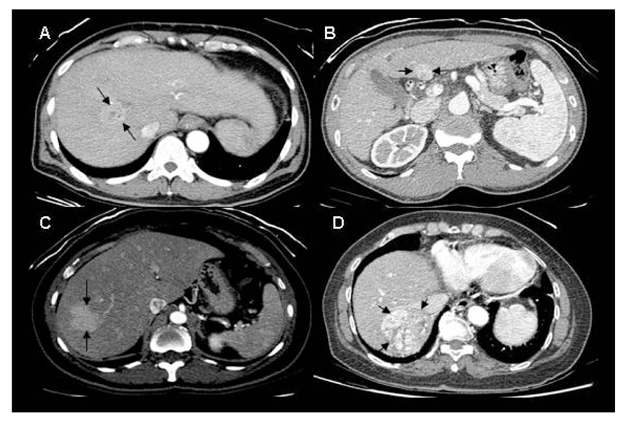
Reference Images for grading visual conspicuity of lesions. Non-visualised lesions were graded 1; A poorly visualised lesion of grade 2 conspicuity (A); An adequately visualised lesion with poor margin delineation of grade 3 conspicuity (B); A lesion of grade 4 conspicuity with good delineation of almost the entire margin (C); A lesion of grade 5 conspicuity with clear demarcation of the entire margin (D).

Subjective assessment of lesion conspicuity was performed independently by three radiologists who were blinded to each other’s findings and with 30, 20 and 6 years of experience in abdominal CT respectively, using a 5-point scale based on a set of standard reference images (Figure 3).

### Statistical Analysis

Statistical analysis was performed using SPSS version 17.0 and the non-parametric Kruskal-Wallis test to examine intergroup differences among the three scan protocols for both the CI and the conspicuity scores. A p value of less than 0.05 was considered to indicate a statistically significant difference.

## RESULTS

Out of 197 patients who underwent 5-phase liver CT from February to October 2008, 120 were excluded (Table 1).

**Table 1:** Reasons for patient exclusion.

**Reasons for patient exclusion**	**Number of patients**
TACE	27
RFA	24
Portal hypertension, thrombosis of portal vein, hepatic vein or vena cava	14
Hepatectomy	11
RFA & TACE	10
Diffuse lesions or lesions > 5cm in size	4
RFA & surgery	3
PTC	1
Liver failure	1
RFA & portal hypertension or portal venous thrombosis	1
RFA & arterioportal shunt	1
TACE & surgery	1
TACE & portal venous thrombosis	1
Transjugular intrahepatic portosystemic shunt & portal hypertension	1
RFA & TACE & surgery	1
RFA & TACE & portal venous thrombosis	1
Technical error	18
**Total**	**120**

**Figure 4:**
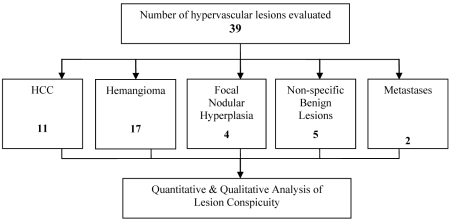
The number and types of hypervascular lesions evaluated.

Among the 77 patients who were included (40 male; 37 female; age 15–90; mean age 59, SD 13.478), 39 hypervascular liver lesions were evaluated (Figure 4). The diagnosis of each lesion was made with a consensus achieved among the three radiologists.

**Figure 5:**
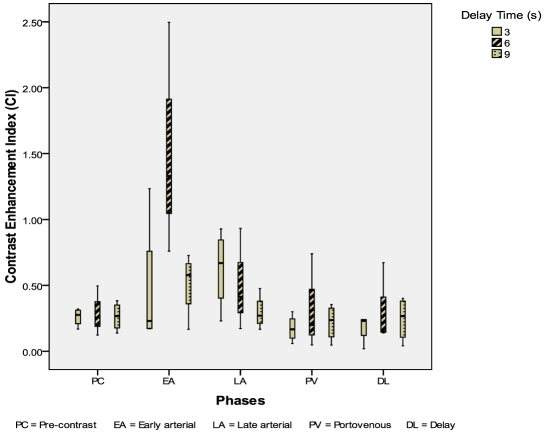
Box and whisker plot illustrating CI for HCC during different scan phases.

**Figure 6:**
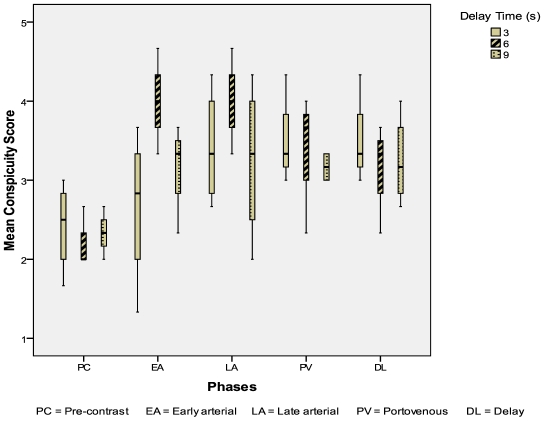
Mean conspicuity scores for HCCs during different scan phases.

Eleven HCC were evaluated: 4 from the 3-second delay group; 3 from the 6-second delay group; 4 from the 9-second delay group. The CIs of HCC were highest in the 6-second delay group during the early arterial phase (p = 0.08) (Figure 5). The mean conspicuity scores of HCC were also higher in the 6-second delay group during both the arterial phases (p = 0.144, p = 0.465) (Figure 6).

**Figure 7:**
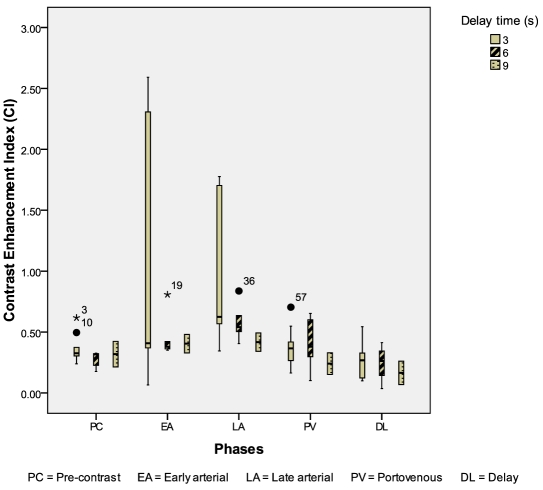
Box and whisker plot illustrating CI for liver haemangioma during different scan phases.

**Figure 8:**
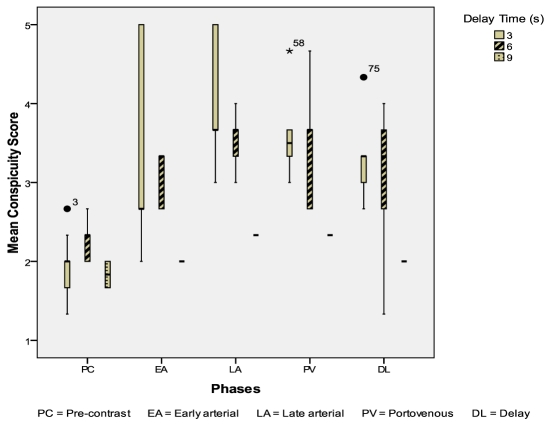
Mean conspicuity scores for liver hemangiomas during different scan phases.

Seventeen liver hemangiomas were evaluated: 10 from the 3-second delay group; 5 from the 6-second delay group; 2 from the 9-second delay group. The CIs of liver hemangiomas were higher in the 3 seconds delay group during the early arterial & late arterial phases (p = 0.824, p = 0.163) (Figure 7). The mean conspicuity scores of liver hemangiomas were also higher in the 3-second delay group during both the arterial phases (p = 0.093, p = 0.033) (Figure 8).

**Figure 9:**
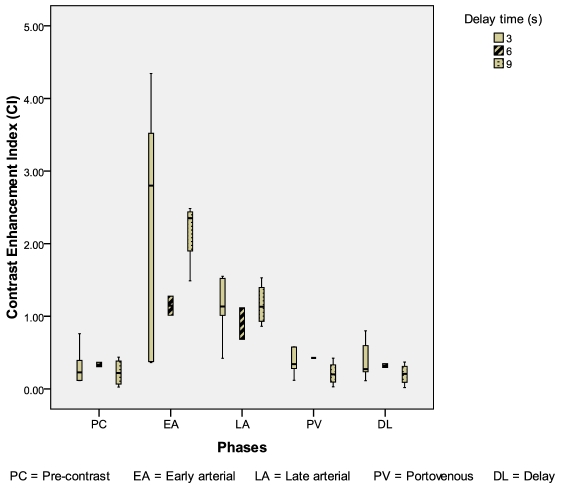
Box and whisker plot illustrating CI for the other hypervascular lesions during different scan phases.

**Figure 10:**
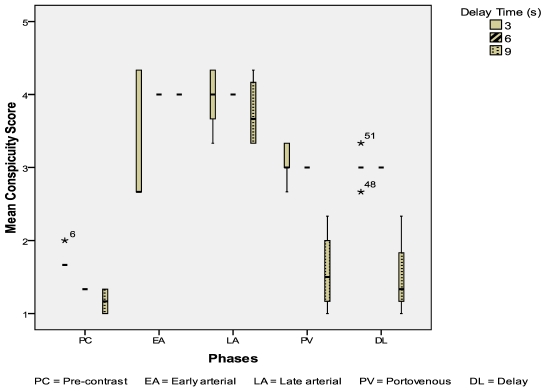
Mean conspicuity scores for the other hypervascular lesions during different scan phases.

The other hypervascular liver lesions which include focal nodular hyperplasia (FNH), the non-specific benign hypervascular lesions and the hypervascular metastases, were analysed together. There were 5 hypervascular lesions in the 3-second group, 2 in the 6-second group and 4 in the 9-second group. The CI was highest in the 3-second group during the early arterial phase (p = 0.499) (Figure 9). The mean conspicuity scores for these hypervascular liver lesions are higher in the 3-second group during both the early arterial and late arterial phases (p = 0.524, p = 0.179) (Figure 10).

## DISCUSSION

The automated bolus tracking technique has been widely recognised to be superior to the fixed-time delay technique in multiphase liver CT, by allowing individualisation and better timing of the scan delay [10–17]. However, in the authors’ institution, five-phase dynamic CT of the liver has been routinely performed using a fixed-time delay technique for the investigation, and follow-up of suspected and known liver lesions. This study aimed to determine the optimal scan delay using the bolus tracking technique for the detection of hypervascular liver lesions.

The three scan protocols using 3-second, 6-second and 9-second scan delays represent an actual delay time of 6 seconds, 9 seconds and 12 seconds, respectively, from the time when threshold enhancements (100 HU) were detected. These protocols were designed based on a previous study by Myeong-Jin Kim et al., which suggested a scan window of 14 to 30 seconds from a 100 HU threshold in the abdominal aorta at the celiac axis level for the detection of hypervascular HCCs. In their study, the duration of each phase scan was 4.5–8.8 seconds and the second arterial phase scan started 6 seconds after the end of the early scan [13]. Therefore, the beginning of the early arterial phase scan ranges from 3.5 seconds to 15.2 seconds from the 100 HU threshold in the abdominal aorta.

In our study, the CIs for HCCs were significantly higher during the early arterial phase scan in the 6-second delay group (p = 0.08). Correspondingly, the mean conspicuity scores for HCCs were highest in the 6-second delay group during both the arterial phases. Given a small sample size of 11 HCCs, a p value of 0.08 inferred a statistically significant difference in a larger study population.

An optimal temporal window of 36–56 seconds in arterial phase imaging had been suggested by Lee et al. for the detection of HCC using a single-detector CT. Frederick et al. suggested a temporal window of less than 45 seconds for the detection of hypervascular lesions [13]. Several other studies on double arterial phase CT using a 4-slice CT found either one of the early or late arterial phase images to be too early or too late for optimal depiction of hypervascular HCCs [7,9,13]. In a study using a 16-slice CT, biphasic arterial phase scan and bolus tracking technique, the optimal scan window in arterial phase imaging for the detection of hypervascular HCC was found to be 14–30 seconds from 100 HU threshold in the abdominal aorta at the celiac axis level [13] In our 6-second delay protocol, the actual temporal delay for the first arterial phase scan was 9 seconds from the 100 HU threshold in the abdominal aorta at the celiac axis level, which was slightly earlier than the temporal window of 14 to 30 seconds suggested by Myeong-Jin Kim et al. [13].

The injection protocols in the two studies were very similar. In the previous study, the contrast dose was adjusted according to body weight [13]. However, a standard injection protocol was applied in our study regardless of patient’s body weight. This is to avoid calculation error in contrast dosage in the setting of a busy radiology department. To overcome the potential limitation caused by contrast dosing on the time to peak enhancement, a relatively high volume (120 ml) and high concentration (370 mg I/ml) contrast media was used. The volume of 120 ml and concentration of 370 mg I/mL consisted of 44,400 mg Iodine, which was equivalent to an iodine dose of 525 mg/kg in an 84 kg patient or 888 mg/kg in a 50 kg patient. In the study by Yumi Yanaga et al. on the optimal contrast dose for depiction of hypervascular HCCs, administration of 450 mg I/kg body weight was considered adequate and a total iodine dose of 525 mg I/kg body weight was considered desirable for excellent depiction of hypervascular HCC [18]. Our injection protocol was adequate to provide a desirable iodine dose in a patient of up to 84 kg in body weight.

High speed injections of up to 7 ml/s had been used in a study on HCC using dynamic 16-MDCT in cine mode by Xiaozhou Ma et al. [19] and an injection rate as high as 10 ml/s had been used by Bader et al. in a liver perfusion study. However, a moderate injection rate at 4 ml/s, which was applied in this study, has been accepted by many researchers [16] in order to avoid patients’ discomfort and to reduce the risk of extravasation. The authors also believe that the high iodine concentration of 370 mg I/mL was able to compensate for the moderate injection rate in achieving adequate conspicuity of hypervascular liver lesions.

The higher CIs of liver hemangiomas in the 3-second delay group corresponded with the higher mean conspicuity scores in the same group during both the arterial phases. The lack of statistical significance of these results may be explained by an unequal patient distribution among the three groups which had occurred by chance during random assignment of patients into one of the three groups prior to the CT examinations. There were 10 out of 17 patients (59%) with liver hemangiomas in the 3-second delay group, 5 patients (29%) in the 6-second delay group and only 2 patients (12%) in the 9-second delay group. The significantly larger sample number in the 3-second delay group compared to the other two groups could have skewed the results to the more favourable CIs and mean conspicuity scores in this group.

Data pooling aims to increase the statistical power in the outcome of analysis. All hypervascular lesions other than HCCs and the classical hemangiomas were evaluated as a group in view of the small number of each of these lesions, which include the FNHs, the non-specific benign hypervascular lesions and the hypervascular metastases. The higher CIs of these lesions which were observed in the 3-second delay group may again be attributed to the unequal patient distribution, of which the majority (45%) was in the 3-second delay group.

In this study, nine out of eleven HCCs were diagnosed on CT, out of which four were further supported by elevated serum AFP levels and one by characteristic MRI findings. Another tumour nodule of 2 cm was diagnosed based on characteristic CT findings, a history of chronic hepatitis B with cirrhosis and an abnormal AFP level. Only one tumour was diagnosed by histopathology examination. According to the American Association for the Study of Liver Diseases (AASLD) guidelines [20] and the unpublished consensus of the European Association for the Study of the Liver experts [21], a diagnosis of HCC can be made when the characteristic HCC profile of intense arterial uptake followed by contrast washout in delayed venous phase is observed in a nodule larger than 2 cm with a cirrhotic liver on a single dynamic imaging technique or on two dynamic imaging techniques in nodules between 1 and 2 cm [20,21]. The Asian Oncology Summit in 2009 recommended diagnosis of HCC based on characteristic image on dynamic CT or MRI regardless of tumour size without tissue biopsy (limited-resource level, level 2a evidence). The sensitivity and specificity of dynamic CT in the diagnosis of HCC was 68% (95% CI 55–80%) and 93% (89–96%); 81% (70–91%) and 85% (77–93%) for dynamic MRI, compared with pathological examination as the reference standard [22]. Although a normal serum AFP concentration (10–20 ng/mL) has been observed in 20% of HCC patients, an AFP concentration > 400 ng/mL in a patient with liver cirrhosis or chronic hepatitis (basic-resource level, level 2a evidence) [22] and a mass larger than 2 cm in a patient with an AFP > 200 ng/dL are considered diagnostic of HCC [23]. A 1–3% risk of tumour seeding during liver biopsy has been reported. Biopsy of potentially operable lesions should therefore be avoided (limited-level resource, level 2a evidence) unless a major diagnostic doubt cannot be resolved with dynamic imaging and measurement of AFP concentration (evidence grade IIa, recommendation grade B) [22, 24]. Tumour location preventing needle insertion, clotting disorders, ascites and false-negative results caused by sampling error or unfeasibility of confidently distinguishing between dysplastic changes and well-differentiated HCC are other recognised limitations of needle biopsy [21].

Liver hemangiomas were diagnosed based on the classical hypodensity similar to that of vessels on non-enhanced CT, peripheral globular enhancement isodense to that of the aorta during the arterial phases followed by centripetal fill-in pattern similar to those of the blood pool during the portal venous phase [25]. FNHs were characterised by hypervascularity during the arterial phases and isodensity in the portovenous and delayed phases. There were five lesions which did not meet the imaging diagnostic criteria of haemangioma or FNH and were unchanged on follow-up imaging. We labelled these lesions as non-specific benign hypervascular lesions. These may represent an atypical haemangioma, FNH or, less commonly, an adenoma. A definitive histopathological diagnosis was not sought because of their benign nature.

In this study, the three different CT protocols which were performed in three patient groups were compared, where the results may be affected by inter-individual variability among the different groups of patients. However, repeating the CT examination in the same patients without clear clinical indication is non-justifiable due to the large radiation dose to patient.

The biggest limitation of this study was the small sample size of each lesion group despite an acceptable size of sample population, especially for the HCC group. Almost all patients with HCCs underwent treatment which potentially altered liver vascularity prior to a follow-up imaging which made them no longer eligible for the study (85%, 112 out of 120). For patients with hemangiomas or other benign lesions, follow-up imaging by ultrasound was preferable rather than CT or MRI.

Lesions larger than 15 cm were excluded in view of the fact that larger size lesions may affect liver vascularity. However, further analysis of data based on lesion’s size, body weight and other factors was not carried out, which may affect the conspicuity of liver lesions due to a small study sample, which is also a limitation in this study.

Thirdly, the lack of pathological proof in lesion diagnoses could be a potential limitation. Exclusion of atypical HCCs or hemangiomas, inclusion of non-HCC hypervascular lesions into the HCC group and inclusion of non-haemangiomas into the haemangioma group had been possible.

Finally, the patients in this study were not classified based on the degree of tumour angioinvasion, background cirrhosis, liver function status and fatty infiltration, which may potentially affect the enhancement pattern and the conspicuity of liver lesions.

## CONCLUSION

The conspicuity of hypervascular HCCs seemed to be better on images obtained during the early arterial phase by using a bolus tracking technique with a scan delay of 6 seconds from the 100 HU threshold in the abdominal aorta at the celiac axis level, i.e. 9-second delay from the initiation of contrast injection. To compare this with delays of 15–30 seconds would require further studies as this imaging is protocol-driven. In keeping with minimising the radiation dose, it is proposed to exclude the pre-contrast phase. To exclude the delayed phase of imaging would require a larger group to be evaluated.
